# Supplementary material for: Creating enzymes and self-sufficient cells for biosynthesis of the non-natural cofactor nicotinamide cytosine dinucleotide
Source: Nat Commun. 2021 Apr 9;12:2116. doi: 10.1038/s41467-021-22357-z (PMC8035330; doi:10.1038/s41467-021-22357-z)
Supplement: Supplementary file 1 — Supplementary Information [file 41467_2021_22357_MOESM1_ESM.pdf]

Supplementary information

Supplementary Figure 1: The crude enzyme activity of suspects in single-site saturation mutation library.

Supplementary Figure 2: The specific activity (S.A.) of suspects in large side-chain amino-acids at multi-site mutation library.

Supplementary Figure 3: The process of constructing the vectors for expression of NcdS.

Supplementary Figure 4: The enzyme-coupled colorimetric assays for activity determination.

Supplementary Figure 5: The crystal structure of wild-type NadD and NcdS-2.

Supplementary Figure 6: The NCD-biosynthesis modular with NMN and CTP supplying.

Supplementary Figure 7: Extracting ions chromatograms of both NAD and NCD in both wild-type *E. coli* and the NCD self-sufficient strains.

Supplementary Table 1: Data-collection and refinement statistics for NcdS-2.

Supplementary Table 2: Mass spectrometry parameters for cofactor detection.

Supplementary Table 3: Strains used in this study.

Supplementary Table 4: Plasmids used in this study.

Supplementary Table 5: Primers used in this study.

Supplementary Table 6: Strategies for constitutive NCD biosynthesis module.

Supplementary Table 7: PCR-strategies for inducible NCD biosynthesis module.

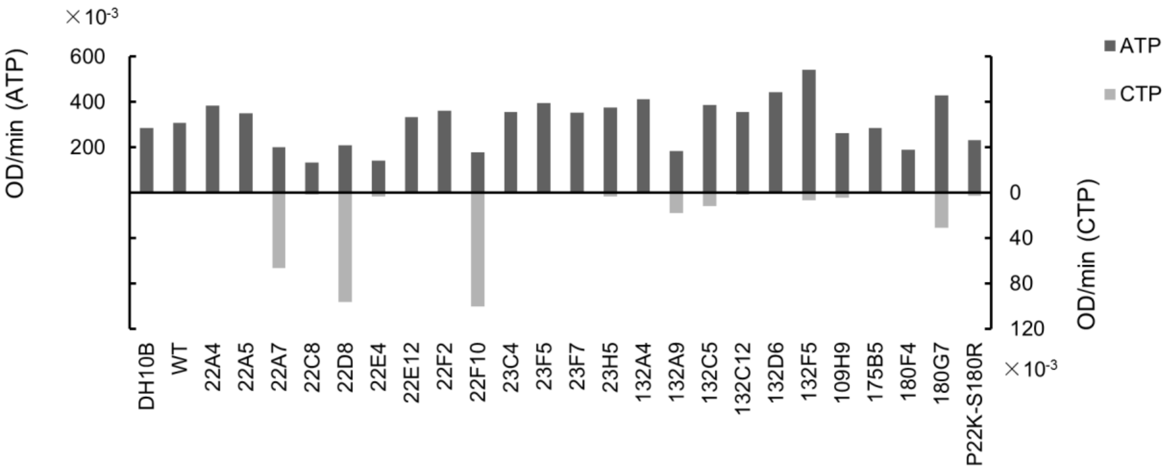

22		D	I	I	K	R	K	R	R	M	K															K
23		V										S	L	Q	H											
132		C														M	K	V	G	N	T					
109		D																				R				
175		P																					T			
180		S																						R	R	R

Supplementary Fig. 1 The crude enzyme activity of suspects in single-site saturation mutation library. The assays were performed using crude extracts and using 100 μM NMN and 2.0 mM ATP or CTP as substrates. DH10B, the host strain; WT, DH10B with wild-type NadD overexpression. The code under the bar represent different mutants in libraries. The natural and mutant amino acids are labeled below. P22K/S180R was a combinatorial mutant.

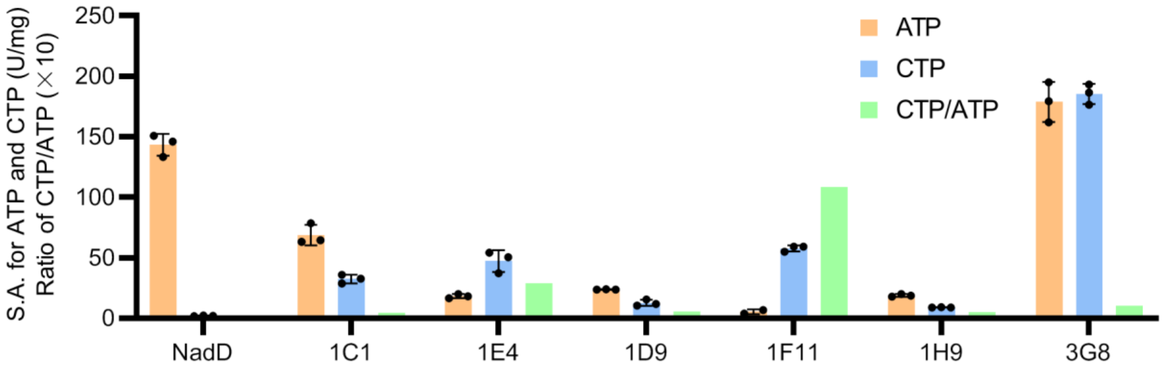

	NadD	1C1	1E4	1D9	1F11	1H9	3G8
22	P	K	K	K	K	K	
23	V						Q
132	C	L	L	L	Q	L	
176	W	L	Y	F	F	Q	E

Supplementary Fig. 2 The specific activity (S.A.) of suspected mutants in large side-chain amino-acids at multi-site mutation library. The natural and mutant amino acids are labeled below each mutant. Assays were done with purified proteins by using 0.1 mM NMN and 2.0 mM ATP or CTP as substrates. Experiments were conducted in triplicates (n = 3), and data are presented as mean values +/- SD.

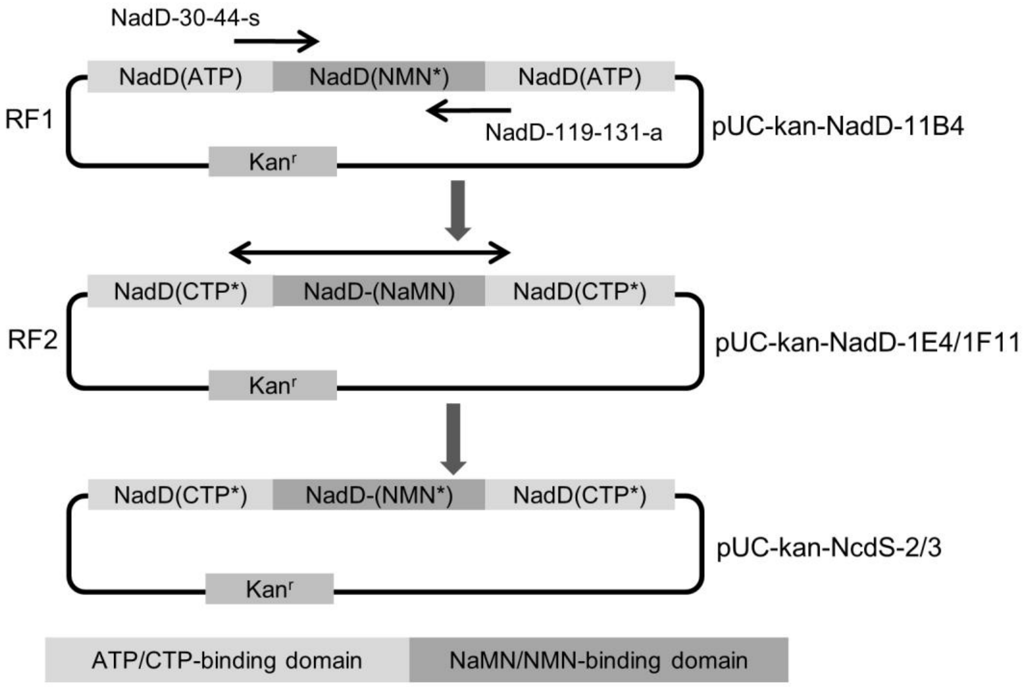

Supplementary Fig. 3 The process of constructing the vectors for expression of NcdS. RF cloning strategy was used to construct the vectors. Primer pairs NadD-30-44-s and NadD-119-131-a were used to amplify the coding sequence of NMN-preferring domain from the template pUC-kan-NadD-11B4. Then the gel-purified fragment was used as mega-primer and pUC-kan-NadD-11E4 or pUC-kan-NadD-1F11 was used as template to amplify the coding sequence of CTP-preferring domain and its vector backbone. The resultant vectors pUC-kan-NcdS-2 and pUC-kan-NcdS-3 were used to express NcdS-2 and NcdS-3, respectively.

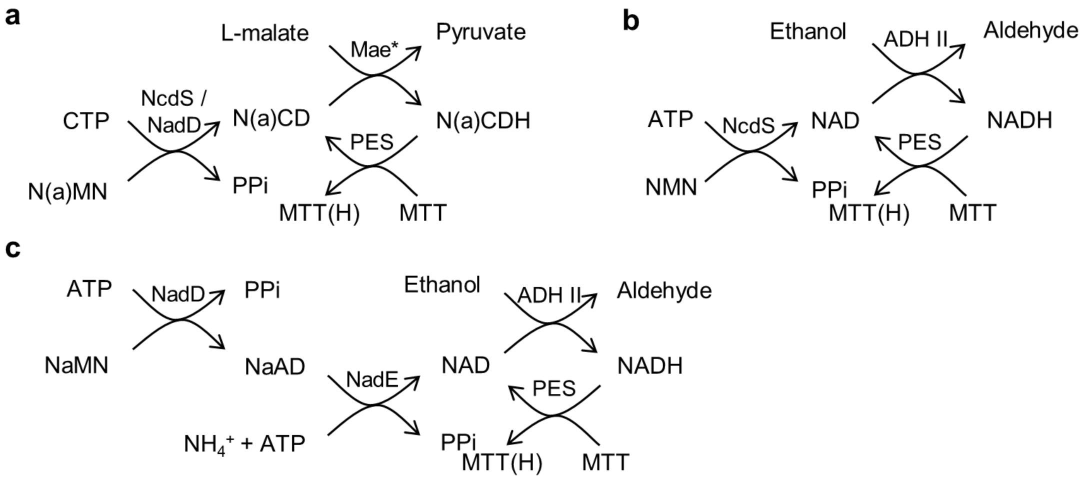

Supplementary Fig. 4 The enzyme-coupled colorimetric assays for activity determination.

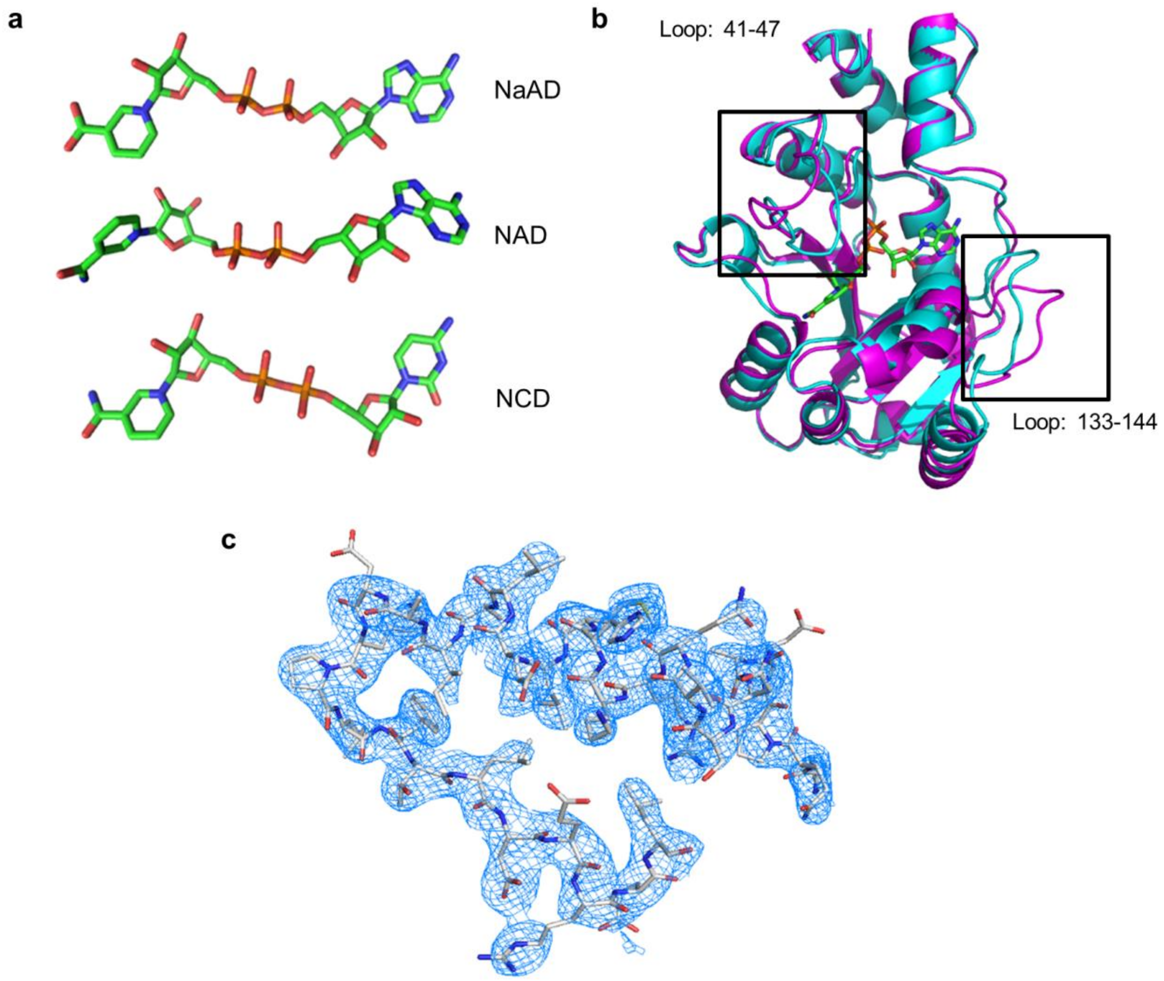

Supplementary Fig. 5 The crystal structure of wild-type NadD and NcdS-2. **a.** The comparison between NaAD, NAD and NCD. **b.** The comparison between wild-type of NadD and NcdS-2. The protein structure was shown as cartoon mode. Wild-type NadD was shown in cyan, NcdS-2 was shown in magenta. Ligands were shown as stick mode. Green, carbon; blue, nitrogen; red, oxygen; orange, phosphorus. **c.** Electron density maps of residue 47 to 77 in NcdS-2. Electron density maps are 2Fo-Fc maps contoured to 1.5.

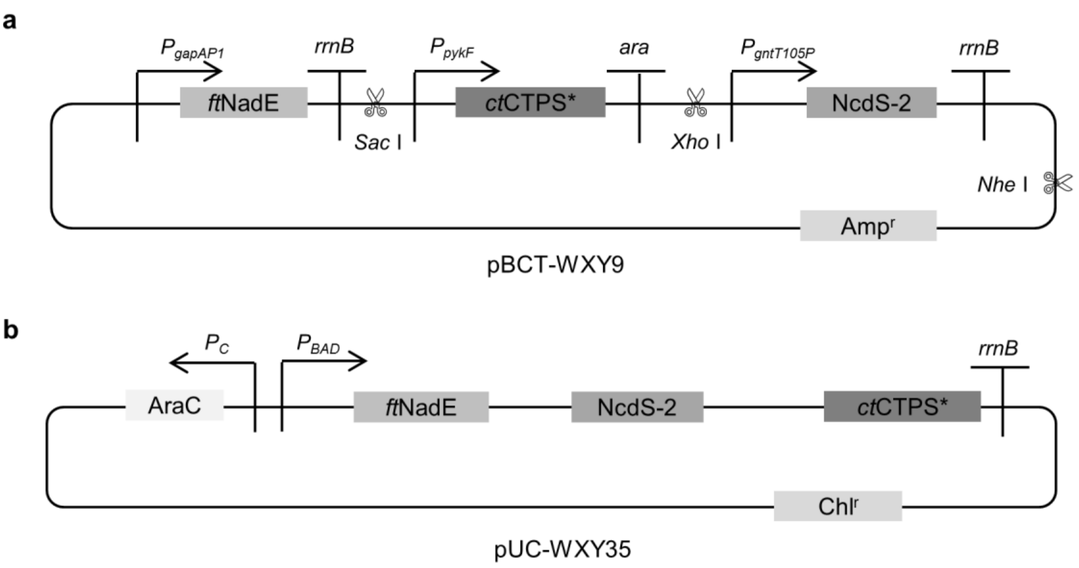

Supplementary Fig. 6 The NCD-biosynthesis modular with NMN and CTP supplying. The modular expressed as constituted (**a**) and regulated by L-arabinose (**b**).

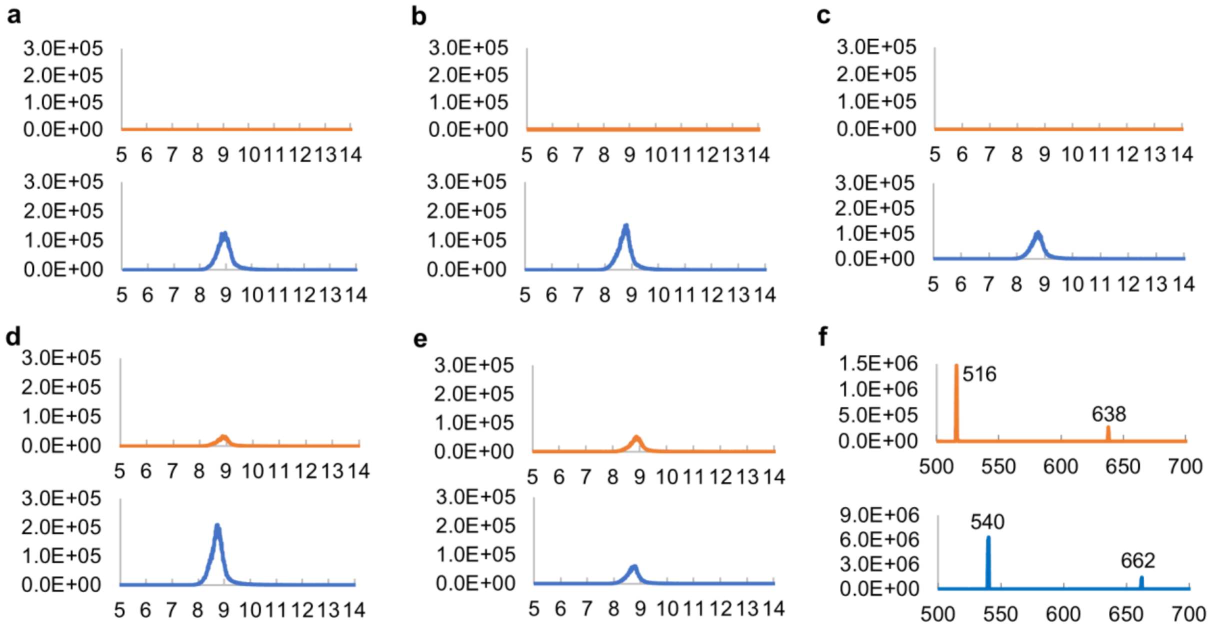

Supplementary Fig. 7 Extracting ions chromatograms of both NAD and NCD in wild-type *E. coli* and the NCD self-sufficient strains. **a.** DH5α. **b.** DH10B. **c.** BW25113(*△ldhA, dld::cat*). **d.** BW25113(*△ldhA, dld::cat*) with 500 μM NAD and 500 μM NCD supplement. **e.** XYC5016. **f.** MS-MS spectrum of NAD and NCD standard. Blue, NAD. Orange, NCD. Characteristic ion pairs 638.000/516.000 and 662.000/540.000 were selected as multiple reaction monitoring ions for NCD and NAD analysis, respectively. The experiments were performed independently twice, with similar results.

Supplementary Table 1. Data-collection and refinement statistics for NcdS-2.

	NcdS-2
**Data collection**	
Space group	$P2_1$
Cell dimensions	
a, *b*, *c* (Å)	84.69, 130.21, 106.74
α, β, γ (°)	90.00, 103.51, 90.00
Resolution (Å)	48.70-3.04 (3.15-3.04) *
R_{sym} or R_{merge}	0.1418 (0.5614)
I / σI	4.5 (1.47)
Completeness (%)	63.36 (40.37)
Redundancy	1.8 (1.7)
**Refinement**	
Resolution (Å)	3.04
No. reflections	27380
R_{work} / R_{free}	0.2317 (0.3471)/0.2361(0.3637)
No. atoms	10189
Protein	10189
Ligand/ion	-
Water	-
B-factors	59.64
Protein	59.64
Ligand/ion	-
Water	-
R.m.s. deviations	
Bond lengths (Å)	0.008
Bond angles (°)	1.32

*Number of xtals for each structure should be noted in footnote. *Values in parentheses are for highest-resolution shell.

Supplementary Table 2. Mass spectrometry parameters for cofactor detection.

Name	Q1	Q3	Dwell time (msec)	Declustering potential	Collision energy
NAD	661.9	540	40	-100	-30
NCD	638	516.2	40	-100	-22

Supplementary Table 3 Strains used in this study.

Strains	Genotype or characteristic	Resource
E. coli strains		
DH10B	F^{-} *endA1 deoR recA1 galE15 galK16 nupG rpsLΔ(lac)X74* φ80*lacZΔM15 araD139 Δ(ara, leu)7697 mcrA Δ(mrr-hsdRMS-mcrBC)* Str^{R} λ^{-}	Invitrogen
DH5α	F-, *φ80d/lacZΔM15*,*Δ(lacZYA-argF)U169*, *deoR*, *recA1*, *endA1*, *hsdR17(rk-, mk+)*, *phoA*, *supE44*, *λ-*, *thi-1*, *gyrA96*, *relA1*	TaKaRa
BW25113(*ΔldhA, dld::cat*)	rrnB3, *ΔlacZ4787, hsdR514, Δ(araBAD)567, Δ(rhaBAD)*568 rph-1, *△ldhA*, *dld::cat*	This study
XYC2002	DH5α / pBCT-PgntT105P-NcdS-2	This study
XYC2008	DH5α / pUC-Para-*Ft*NadE-c-his-NcdS-2-*Ct*CTPS*	This study
XYC2009	DH5α / pUC-Para-NcdS-2	This study
XYC2010	DH5α / pUC-Para-*Ct*CTPS*-NcdS-2	This study
XYC2012	DH5α / pUC-Para-*Ft*NadE-c-his-NcdS-2	This study
XYC2013	DH5α / pBCT-PgapAP2-*Ft*NadE-PpykF-*Ct*CTPS*-PgntT105P-NcdS-2	This study
XYC2017	DH5α / pUC-P15A-Para-*Ft*NadE-c-his-NcdS-2-*Ct*CTPS*	This study
XYC5008	BW25113(*ΔldhA, dld::cat*) / pUC-Para-*Ft*NadE-c-his-NcdS-2-*Ct*CTPS*	This study
XYC5016	BW25113(*ΔldhA, Δdld, arsB::Para-FtnadE-c-his-ncds-2-Ctctps**)	This study
XYC5017	BW25113(*ΔldhA, dld::cat*) / pUC-P15A-Para-*Ft*NadE-c-his-NcdS-2-*Ct*CTPS*	This study
XYC6017	XZ654 / pUC-P15A-Para-*Ft*NadE-c-his-NcdS-2-*Ct*CTPS*	This study
XYC8017	DH18B / pUC-P15A-Para-*Ft*NadE-c-his-NcdS-2-*Ct*CTPS*	This study
XYD042	XYC5016 / pUC-Plac-Ldh*-rbs-Mae*	This study
XYD046	BW25113(*ΔldhA, dld::cat*) / pUC-Plac-Ldh*-rbs-Mae*	This study

Supplementary Table 4 Plasmids used in this study.

Plasmids		Genotype or characteristic	Resource
	pBCTA	*lacI, pBR322* ori*, bla, gapAP1* promoter	Our lab
	pBCTB	*lacI, pBR322* ori*, bla, gntT105P* promoter	Our lab
	pK	pUC18, *bla::kan*	Our lab
	pK-Mae*-Ldh*	pUC18, Mae* with *lac* promoter, Ldh* with *lac* promoter, *bla::kan*	Our lab
	pK-Ldh-V152R/N213C	pUC18, Ldh-V152R/N213C expression, *bla::kan*	Our lab
	pODC29-*Ft*NadE	pET-derived, *Ft*NadE with 6×his-tag expression	[1]
	pUC57-*Ct*CTPS*	pUC57 with *Ct*CTPS*-6×his expression, *bla*	This study
pUC-WXY1	pUC-kan-NadD	pK with NadD-6×his expression	[2]
pUC-WXY3	pUC-kan-NadD-mRFP	pK with NadD-RFP-6×his expression	[2]
pUC-WXY4	pUC-kan-NadD-11B4	pK with NadD-Y84V/Y118D-6×his *expression*	[2]
pUC-WXY14	pUC-kan-NcdS-2	pK with NcdS-2-6×his expression	This study
pUC-WXY19	pUC-PolB-DedA	pUC18 with *lacZ* replaced by *ara* operon	This study
pUC-WXY22	pUC-chl-Para-*Ft*NadE-c-his	pUC-polB-DedA with *Ft*NadE-6×his expression, *ara* operon, *bla::cat*	This study
pUC-WXY23	pUC-chl-Para-*Ct*CTPS*	pUC-polB-DedA with *Ct*CTPS*-6×his expression, *ara* operon	This study
pUC-WXY27	pUC-Para-*Ct*CTPS*-rbs	pUC18 with *lacZ* replaced by *ara* operon and *Ct*CTPS*-6×his coding sequence	This study
pUC-WXY30	pUC-Para-NcdS-2	pUC-PolB-DedA with NadD-P22K/Y84V/Y118D/C132L/W176Y-6×his expression, *ara* operon	This study
pUC-WXY32	pUC-Para-*Ft*NadE-c-his-NcdS-2	pUC-polB-DedA with *Ft*NadE-6×his and NcdS-2-6×his expression, *ara* operon	This study
pUC-WXY33	pUC-Para-*Ct*CTPS*-NcdS-2	pUC-polB-DedA with *Ct*CTPS*-6×his and NcdS-2-6×his expression, *ara* operon	This study
pUC-WXY35	pUC-Para-*Ft*NadE-c-his-NcdS-2-*Ct*CTPS*	pUC-polB-DedA with *Ft*NadE-6×his, NcdS-2-6×his and *Ct*CTPS*-6×his expression, *ara* operon	This study
pUC-WXY36	pUC-chl-PpykF-*Ct*CTPS*	pUC18, *Ct*CTPS with *pykF* promoter, *bla::cat*	This study
pUC-WXY63	pUC-P15A-Para-*Ft*NadE-c-his-NcdS-2-*Ct*CTPS*	pUC-WXY34 with *pBR322* ori replaced by P15A ori	This study
pUC-WXY65	pUC-Plac-Ldh*-rbs-Mae*	pK, Ldh* and Mae* with lac operon	This study
pBCT-WXY1	pBCT-PgapAP1-*Ft*NadE	pBCTA with 6×His-*Ft*NadE inserted at *Nde* I and *Kpn* I site, *bla*, *gapAP1* promoter	This study
pBCT-WXY4	pBCT-PgntT105P-NcdS-2	pBCTB, NadD-P22K/Y84V/Y118D/C132L/W176Y-6×his inserted at *EcoR* I and *BamH* I site, *bla*, *gntT105P* promoter	This study
pBCT-WXY7	pBCT-PgapAP1-*Ft*NadE-PpykF-*Ct*CTPS*	*Ft*NadE-6×his with *gapAP1* promoter, *Ct*CTPS-6×his with *pykF* promoter, amp	This study
pBCT-WXY9	pBCT-PgapAP2-*Ft*NadE-PpykF-*Ct*CTPS*-PgntT105P-NcdS-2	pBCTB, *Ft*NadE-6×his with *gapAP1* promoter, *Ct*CTPS-6×his with *pykF* promoter, NcdS-2-6×his with *gntT105P* promoter	This study

Supplementary Table 5. Primers used in this study.

Primer name	Primer sequence (5’→3’)
NadD-30-44-s	ggtctgacgcgggtcacaatcatccctaataatgttcctcc
NadD-119-131-a	gacgatcaaatgtgcattgtcgagtatcgtttcgtattc
pBCTB-NadD-s	ccagcgcaaatgaataaggagatagaattcatgaaatctttacaggctctgtttgg
pBCTB-NadD-a	gcaggtcgactctagaggatccttagtggtggtggtggtggtggcgatacaagccttgttgg
pBCTA-*Ft*NadE-NdeI-s	ccttttattcactaacaaatagctggtggaacatatgcaccaccaccaccaccacaaaatagttaaag
pBCTA-*Ft*NadE-KpnI-a	gcatgcctgcaggtcgactctagaggtaccttagaaattaggagttaaagc
pPykF-CTPS-s	ccatacccgttttttggatggagtgaaacgtctttatacctatttatcatgccaactatcagc
pPykF-CTPS-a	ggtcaggaagatgcttttgaagctcatgacagtcttagtctttaagttgagaagg
pUC-PolB-DedA-s	ggtgatgacggtgaaaacctctgacacatgcagcggtgataaaccgctccatcagatagcgttctgg
pUC-PolB-DedA-a	gcttccggctcgtatgttgtgtggaattgtgccagaaagaaatccagtcgcccatcaagcagcc
pUC-Para-NcdS-s	cgttttttggatggagtgaaacgatgaaatctttacaggctctgtttgg
pUC-Para-NcdS-a	ggctgtggttttatacagtcattagtggtggtggtggtggtggcgatacaagc
Para-*Ft*NadE-c-his-s	ccatacccgttttttggatggagtgaaacgatgaaaatagttaaagattttagtcc
Para-*Ft*NadE-c-his-a	cctggtttcgtttgattggctgtggttttatacagtcattagtggtggtggtggtggtggaaattagg
Para-*Ct*CTPS*-s	cgttttttggatggagtgaaacgatgagcttcaaaagcatcttcctgacc
Para-*Ct*CTPS*-a	ggctgtggttttatacagtcattagtggtggtggtgatggtgacgatgttcg
Para-*Ct*CTPS*-rbs-s	gcatccggcacgaaggagtcaacatgagcttcaaaagcatcttcctgacc
Para-*Ct*CTPS*-rbs-a	ggtgaaaacctctgacacatgcagcttagtggtggtggtgatggtgacg
*Ft*NadE-c-his-NcdS-s	gctttaactcctaatttccaccaccaccaccaccactagggacacgataatgaaatctttacaggctctgtttgg
*Ct*CTPS*-NcdS-s	cgaaccacgaacatcgtcaccatcaccaccaccactaaggacacgataatgaaatctttacaggctctgtttgg
NcdS-*Ct*CTPS*-s	gcttgtatcgccaccaccaccaccaccactaagtagccgcatccggtatgtaacgcctgatgc
NcdS-*Ct*CTPS*-a	cgtttgattggctgtggttttatacagtcattagtggtggtggtgatggtgacg
NadD-P22X-s	ggtgcactatggtcatctaaaannkgtggaaacgctggcgaatttgattgg
NadD-V23X-s	ggtgcactatggtcatctaaaacccnnkgaaacgctggcgaatttgattgg
NadD-I105X-s	ggacgtgccgctggcgtttnnkattggtcaggattcactgctgacc
NadD-G107X-s	cgtgccgctggcgtttattattnnkcaggattcactgctgacctttcc
NadD-D109X-s	gctggcgtttattattggtcagnnktcactgctgacctttccgacctgg
NadD-C132X-s	cgacaatgcacatttgatcgtcnnkcggcgtccaggttacccacttgaaatgg
NadD-R133X-s	cgacaatgcacatttgatcgtctgtnnkcgtccaggttacccacttgaaatgg
NadD-T174X-a	ggtcgccgagatgttaaaccacggmnnttcagccagataaattttacc
NadD-P175X-a	ggtcgccgagatgttaaaccamnncgtttcagccagataaattttacc
NadD-W176X-a	ggatgatggtcgccgagatgttaaamnncggcgtttcagccagataaattttacc
NadD-N178X-a	ggatgatggtcgccgagatmnnaaaccacggcgtttcagccagataaattttacc
NadD-S180X-a	gcaaacgttcgcggatgatggtcgcmnngatgttaaaccacggcgtttcagc
NadD-S180R-a	gcaaacgttcgcggatgatggtcgcacggatgttaaaccacggcgtttcagc
NadD-C132-6-s-1	cgacaatgcacatttgatcgtcckccggcgtccaggttacccacttgaaatgg
NadD-C132-6-s-2	cgacaatgcacatttgatcgtcuwucggcgtccaggttacccacttgaaatgg
NadD-C132-6-s-3	cgacaatgcacatttgatcgtcsaacggcgtccaggttacccacttgaaatgg
NadD-W176-6-a-1	ggatgatggtcgccgagatgttaaagmgcggcgtttcagccagataaattttacc
NadD-W176-6-a-2	ggatgatggtcgccgagatgttaaaawacggcgtttcagccagataaattttacc

NadD-W176-6-a-3	ggatgatggtcgccgagatgttaaattscggcgtttcagccagataaattttacc
NadD-S180-6-a-1	gcaaacgttcgcggatgatggtcgcgmggatgttaaaccacggcgtttcagc
NadD-S180-6-a-2	gcaaacgttcgcggatgatggtcgcawagatgttaaaccacggcgtttcagc
NadD-S180-6-a-3	gcaaacgttcgcggatgatggtcgcttsgatgttaaaccacggcgtttcagc
NadD-A172T174-6-a-11	ggtcgccgagatgttaaaccacgggmgttcgmgcagataaattttaccggcagg
NadD-A172T174-6-a-12	ggtcgccgagatgttaaaccacgggmgttcawacagataaattttaccggcagg
NadD-A172T174-6-a-13	ggtcgccgagatgttaaaccacgggmgttcttscagataaattttaccggcagg
NadD-A172T174-6-a-21	ggtcgccgagatgttaaaccacggawattcgmgcagataaattttaccggcagg
NadD-A172T174-6-a-22	ggtcgccgagatgttaaaccacggawattcawacagataaattttaccggcagg
NadD-A172T174-6-a-23	ggtcgccgagatgttaaaccacggawattcttscagataaattttaccggcagg
NadD-A172T174-6-a-31	ggtcgccgagatgttaaaccacggttsttcgmgcagataaattttaccggcagg
NadD-A172T174-6-a-32	ggtcgccgagatgttaaaccacggttsttcawacagataaattttaccggcagg
NadD-A172T174-6-a-33	ggtcgccgagatgttaaaccacggttsttcttscagataaattttaccggcagg
NadD-V23L26-6-s-11	gcactatggtcatctaaaacccckcgaaacgckcgcgaatttgattggtctgacg
NadD-V23L26-6-s-12	gcactatggtcatctaaaacccckcgaaacgtwtgcgaatttgattggtctgacg
NadD-V23L26-6-s-13	gcactatggtcatctaaaacccckcgaaacgsaagcgaatttgattggtctgacg
NadD-V23L26-6-s-21	gcactatggtcatctaaaaccctwtgaaacgckcgcgaatttgattggtctgacg
NadD-V23L26-6-s-22	gcactatggtcatctaaaaccctwtgaaacgtwtgcgaatttgattggtctgacg
NadD-V23L26-6-s-23	gcactatggtcatctaaaaccctwtgaaacgsaagcgaatttgattggtctgacg
NadD-V23L26-6-s-31	gcactatggtcatctaaaacccsaagaaacgckcgcgaatttgattggtctgacg
NadD-V23L26-6-s-32	gcactatggtcatctaaaacccsaagaaacgtwtgcgaatttgattggtctgacg
NadD-V23L26-6-s-33	gcactatggtcatctaaaacccsaagaaacgsaagcgaatttgattggtctgacg
NadD-P22V23-6-s-11	ggtgcactatggtcatctaaaackcckcgaaacgctggcgaatttgattgg
NadD-P22V23-6-s-12	ggtgcactatggtcatctaaaackctwtgaaacgctggcgaatttgattgg
NadD-P22V23-6-s-13	ggtgcactatggtcatctaaaackcsaagaaacgctggcgaatttgattgg
NadD-P22V23-6-s-21	ggtgcactatggtcatctaaaatwtckcgaaacgctggcgaatttgattgg
NadD-P22V23-6-s-22	ggtgcactatggtcatctaaaatwttwtgaaacgctggcgaatttgattgg
NadD-P22V23-6-s-23	ggtgcactatggtcatctaaaatwtsaagaaacgctggcgaatttgattgg
NadD-P22V23-6-s-31	ggtgcactatggtcatctaaaasaackcgaaacgctggcgaatttgattgg
NadD-P22V23-6-s-32	ggtgcactatggtcatctaaaasaatwtgaaacgctggcgaatttgattgg
NadD-P22V23-6-s-33	ggtgcactatggtcatctaaaasaasaagaaacgctggcgaatttgattgg
NadD-T174S180-6-a-11	ggatgatggtcgcgmggatgttaaaccacgggmgttcagccagataaattttacc
NadD-T174S180-6-a-12	ggatgatggtcgcgmggatgttaaaccacggawattcagccagataaattttacc
NadD-T174S180-6-a-13	ggatgatggtcgcgmggatgttaaaccacggttsttcagccagataaattttacc
NadD-T174S180-6-a-21	ggatgatggtcgctwtgatgttaaaccacgggmgttcagccagataaattttacc
NadD-T174S180-6-a-22	ggatgatggtcgctwtgatgttaaaccacggawattcagccagataaattttacc
NadD-T174S180-6-a-23	ggatgatggtcgctwtgatgttaaaccacggttsttcagccagataaattttacc
NadD-T174S180-6-a-31	ggatgatggtcgcttsgatgttaaaccacgggmgttcagccagataaattttacc
NadD-T174S180-6-a-32	ggatgatggtcgcttsgatgttaaaccacggawattcagccagataaattttacc
NadD-T174S180-6-a-33	ggatgatggtcgcttsgatgttaaaccacggttsttcagccagataaattttacc

Supplementary Table 6. Strategies for constitutive NCD biosynthesis module.

Primer1 / Primer2	Template1	Template2	Product
pBCTA-*Ft*NadE-NdeI-s/pBCTA-*Ft*NadE-KpnI-a	pODC-*Ft*NadE	pBCTA	pBCT-PgapAP1-*Ft*NadE
pBCTB-NadD-s / pBCTB-NadD-a	pUC-kan-NcdS-2	pBCTB	pBCT-PgntT105P-NcdS-2
pPykF-CTPS-s / pPykF-CTPS-a	DH10BgDNA	pUC-chl-Para-*Ct*CTPS*	pUC-chl-PpykF-*Ct*CTPS*

Supplementary Table 7. PCR-strategies for inducible NCD biosynthesis module.

Primer1 / Primer2	Template plasmids	Target plasmids	Result Plasmids
pUC-PolB-DedA-s / pUC-PolB-DedA-a	DH10BgDNA	pUC18	pUC-PolB-DedA
pUC-Para-NcdS-s / pUC-Para-NcdS-a	pUC-kan-NcdS-2	pUC-PolB-DedA	pUC-Para-NcdS-2
Para-*Ft*NadE-c-his-s / Para-*Ft*NadE-c-his-a	pODC-*Ft*NadE	pUC-PolB-DedA	pUC-chl-Para-*Ft*NadE-c-his
Para-*Ct*CTPS*-s / Para-*Ct*CTPS*-a	pUC57-*Ct*CTPS*	pUC-PolB-DedA	pUC-chl-Para-*Ct*CTPS*
Para-*Ct*CTPS*-rbs-s / Para-*Ct*CTPS*-rbs-a	pUC57-*Ct*CTPS*	pUC-PolB-DedA	pUC-Para-*Ct*CTPS*-rbs
*Ft*NadE-c-his-NcdS-s / pUC-Para-NcdS-a	pUC-Para-NcdS-2	pUC-chl-Para-*Ft*NadE-c-his	pUC-Para-*Ft*NadE-c-his-NcdS-2
*Ct*CTPS*-NcdS-s / pUC-Para-NcdS-a	pUC-Para-NcdS-2	pUC-chl-Para-*Ct*CTPS*	pUC-Para-*Ct*CTPS*-NcdS-2
NcdS-*Ct*CTPS*-s / NcdS-*Ct*CTPS*-a	pUC-Para-*Ct*CTPS*-rbs	pUC-Para-*Ft*NadE-c-his-NcdS-2	pUC-Para-*Ft*NadE-c-his-NcdS-2-*Ct*CTPS*

Supplementary reference

1. Sorci, L. *et al.* Nicotinamide mononucleotide synthetase is the key enzyme for an alternative route of NAD biosynthesis in *Francisella tularensis*. *Proc. Natl. Acad. Sci. USA* **106**, 3083-3088 (2009).

2. Wang, X. *et al.* Engineering *Escherichia coli* Nicotinic Acid Mononucleotide Adenylyltransferase for Fully Active Amidated NAD Biosynthesis. *Appl. Environ. Microbiol.* **83**, e00692-17 (2017).
