# Supplementary material for: Creating enzymes and self-sufficient cells for biosynthesis of the non-natural cofactor nicotinamide cytosine dinucleotide
Source: Nat Commun. 2021 Apr 9;12:2116. doi: 10.1038/s41467-021-22357-z (PMC8035330; doi:10.1038/s41467-021-22357-z)
Supplement: Supplementary file 2 — Reporting Summary [file 41467_2021_22357_MOESM2_ESM.pdf]

nature research

Corresponding author(s): Zongbao K. Zhao
Last updated by author(s): Mar 9, 2021

Reporting Summary

Nature Research wishes to improve the reproducibility of the work that we publish. This form provides structure for consistency and transparency in reporting. For further information on Nature Research policies, see our Editorial Policies and the Editorial Policy Checklist.

Statistics

For all statistical analyses, confirm that the following items are present in the figure legend, table legend, main text, or Methods section.

n/a	Confirmed	
☐	☒	The exact sample size (n) for each experimental group/condition, given as a discrete number and unit of measurement
☐	☒	A statement on whether measurements were taken from distinct samples or whether the same sample was measured repeatedly
☒	☐	The statistical test(s) used AND whether they are one- or two-sided<br>*Only common tests should be described solely by name; describe more complex techniques in the Methods section.*
☒	☐	A description of all covariates tested
☒	☐	A description of any assumptions or corrections, such as tests of normality and adjustment for multiple comparisons
☐	☒	A full description of the statistical parameters including central tendency (e.g. means) or other basic estimates (e.g. regression coefficient) AND variation (e.g. standard deviation) or associated estimates of uncertainty (e.g. confidence intervals)
☒	☐	For null hypothesis testing, the test statistic (e.g. F, t, r) with confidence intervals, effect sizes, degrees of freedom and P value noted<br>*Give P values as exact values whenever suitable.*
☒	☐	For Bayesian analysis, information on the choice of priors and Markov chain Monte Carlo settings
☒	☐	For hierarchical and complex designs, identification of the appropriate level for tests and full reporting of outcomes
☒	☐	Estimates of effect sizes (e.g. Cohen's d, Pearson's r), indicating how they were calculated

Our web collection on statistics for biologists contains articles on many of the points above.

Software and code

Policy information about availability of computer code

Data collection	Diffraction data for NcdS-2 were collected at beam line BL18U1 of the Shanghai Synchrotron Radiation Facility. The intracellular NAD and NCD were analyzed using an Acchrom high-performance liquid chromatography system S6000 (Beijing, China). LC-MS/MS analysis was performed on an ACQUITY UPLC System hyphened to an AB Sciex Qtrap 5500 instrument. All the activity data were analyzed using BIO-TEK PowerWave XS, BIO-TEK SYNERGY H1 and Thermo EVOLUTION 220.
Data analysis	PyMol (v0.99rc6), HKL2000 (v715-linux-x86_64), COOT (v0.8.9), PHENIX (v1.3_2998), MOE package (v2018.01), Empower3 (v2010.3471-c), KC JrWin (v1.41.6), Gen CHS (v3.00), Thermo INSIGHT (v2.0.459)

For manuscripts utilizing custom algorithms or software that are central to the research but not yet described in published literature, software must be made available to editors and reviewers. We strongly encourage code deposition in a community repository (e.g. GitHub). See the Nature Research guidelines for submitting code & software for further information.

Data

Policy information about availability of data

All manuscripts must include a data availability statement. This statement should provide the following information, where applicable:

- Accession codes, unique identifiers, or web links for publicly available datasets
- A list of figures that have associated raw data
- A description of any restrictions on data availability

Coordinates and structure data of NcdS-2 have been deposited in the Protein Data Bank with the accession code 6KH2 [https://www.rcsb.org/structure/6KH2]. Coordinates and structure data of EcNadD are available in the Protein Data Bank with the accession code 1K4M [https://www.rcsb.org/structure/1K4M]. The gene and protein used in this study are available in NCBI with the accession codes as follows: EcNadD, QJZ11375 [https://www.ncbi.nlm.nih.gov/protein/QJZ11375.1/]; FtNadE, EDZ90683 [https://www.ncbi.nlm.nih.gov/gene/60806288]; CtCTPS, AAA80195 [https://www.ncbi.nlm.nih.gov/protein/AAA80195.1/]; EcMae, NP415996

[https://www.ncbi.nlm.nih.gov/protein/NP_415996.2/]; LhLdh, WP003628108 [https://www.ncbi.nlm.nih.gov/protein/WP_003628108.1/]. The source data for Fig. 4d, Fig. 4e, Fig. 5b, Fig. 5c, Fig. 5d, Fig. S1 and Fig. S2 are provided as a Source Data file. All other data are available from the corresponding author upon request.

Field-specific reporting

Please select the one below that is the best fit for your research. If you are not sure, read the appropriate sections before making your selection.

- [x] Life sciences
- [ ] Behavioural & social sciences
- [ ] Ecological, evolutionary & environmental sciences

For a reference copy of the document with all sections, see nature.com/documents/nr-reporting-summary-flat.pdf

Life sciences study design

All studies must disclose on these points even when the disclosure is negative.

Sample size	All samples are conducted with three replicates. Choice of sample sizes guided by established precedents from leading works (DOI: 10.1038/NCHEM.2927) in the field.
Data exclusions	No data were excluded.
Replication	The identification and quantification of intracellular cofactors, enzyme activity determination and organic acid production were repeated at least once, with similar results. Kinetic determination of purified protein were conducted with three replicates. In order to reduce the workload, library screening was done once, and we conducted activity assays of purified suspected hits for further confirmation. Molecular docking results were computationally simulated, and the optimal conformation was predicted by scoring.
Randomization	Not applicable, as no experimental groups were generated.
Blinding	Not applicable, as no experimental groups were generated.

Reporting for specific materials, systems and methods

We require information from authors about some types of materials, experimental systems and methods used in many studies. Here, indicate whether each material, system or method listed is relevant to your study. If you are not sure if a list item applies to your research, read the appropriate section before selecting a response.

Materials & experimental systems

n/a	Involved in the study	
[x]	[ ]	Antibodies
[x]	[ ]	Eukaryotic cell lines
[x]	[ ]	Palaeontology and archaeology
[x]	[ ]	Animals and other organisms
[x]	[ ]	Human research participants
[x]	[ ]	Clinical data
[x]	[ ]	Dual use research of concern

Methods

n/a	Involved in the study	
[x]	[ ]	ChIP-seq
[x]	[ ]	Flow cytometry
[x]	[ ]	MRI-based neuroimaging
